# Difference in Activity in the Supplementary Motor Area Depending on Limb Combination of Hand–Foot Coordinated Movements

**DOI:** 10.3389/fnhum.2016.00499

**Published:** 2016-10-04

**Authors:** Kento Nakagawa, Saeko Kawashima, Nobuaki Mizuguchi, Kazuyuki Kanosue

**Affiliations:** ^1^Faculty of Sport Sciences, Waseda UniversityTokorozawa, Japan; ^2^Japan Society for the Promotion of ScienceTokyo, Japan; ^3^Graduate School of Arts and Sciences, The University of TokyoTokyo, Japan

**Keywords:** interlimb coordination, fMRI, limb combination, supplementary motor area, percent signal change

## Abstract

Periodic interlimb coordination shows lower performance when the ipsilateral hand and foot (e.g., right hand and right foot) are simultaneously moved than when the contralateral hand and foot (e.g., right hand and left foot) are simultaneously moved. The present study aimed to investigate how brain activity that is related to the dependence of hand–foot coordination on limb combination, using functional magnetic imaging. Twenty-one right-handed subjects performed periodic coordinated movements of the ipsilateral or contralateral hand and foot in the same or opposite direction in the sagittal plane. Kinematic data showed that performance was lower for the ipsilateral hand–foot coordination than for the contralateral one. A comparison of brain activity between the same and opposite directions showed that there was a greater activation of supplementary motor area for ipsilateral hand–foot coordination as compared to that seen during contralateral hand–foot coordination. We speculate that this might reflect a difference in the degree of inhibition of the neural circuit that disrupts opposite directional movements between ipsilateral and contralateral hand–foot coordinated movements.

## Introduction

Simultaneous movements of two limbs in the sagittal plane are strongly constrained by movement direction. For example, when subjects try to move their right hand and right foot periodically, opposite directional movements are more variable and less accurate than same directional movements (*directional constraint*; Baldissera et al., 1982; Carson et al., 1995; Muraoka et al., 2013; Nakagawa et al., 2013). Previous studies involving the directional constraint have mainly focused on the effects movement frequency (Carson et al., 1995) and feedback information (Swinnen et al., 1995). Interestingly, some previous studies have also shown that the magnitude of the directional constraint changes depending upon the particular limb combination (Kelso and Jeka, 1992; Swinnen et al., 1995; Hiraga et al., 2004; Meesen et al., 2006; Fujiyama et al., 2013; Nakagawa et al., 2015). That is, coordinated movements of *ipsilateral* upper and lower limbs (e.g., right hand and right foot) show a prominent directional constraint while those of *contralateral* upper and lower limbs (e.g., right hand and left foot) are less prominent. These behavioral difference have been confirmed, but the neural basis as to why the directional constraint of hand–foot coordination are dependent upon limb combination has remained unclear. If the neural mechanisms underlying the effects of limb combination become clear, it would contribute to an overall understanding of interlimb coordination. Therefore, it is important to clarify the brain regions that are associated with the effect of limb combination on hand–foot coordination. Knowledge of differences in the neural basis associated with ipsilateral and contralateral coordination would also aid in understanding more complicated interlimb coordination involving multi limb bilateral movements such as gait (Zehr and Duysens, 2004; Sousa et al., 2013a,b; Sousa and Tavares, 2015)

Brain activity during hand–foot coordination has been examined utilizing brain imaging technique (Debaere et al., 2001; Heuninckx et al., 2005, 2008; Rocca et al., 2007). Debaere et al. (2001) suggest that extra activation of the supplementary motor area (SMA) in the ipsilateral hand–foot coordination in the opposite direction as compared to the same direction. Other studies also showed that the higher motor cortices including the SMA, premotor area (PMA), and the cerebellum, are more activated for ipsilateral hand–foot coordinated movements in the opposite direction than in the same direction (Heuninckx et al., 2005; Van Impe et al., 2009). Thus, the secondary motor areas (SMA and PMA) or the cerebellum may play a key role in determining the stability and accuracy of interlimb coordinated movements under different conditions. However, the tasks utilized in these studies involved only the ipsilateral upper and lower limbs. Therefore, it remains unclear as to whether brain activity is different between ipsilateral and contralateral hand–foot coordinated movements. Thus, in the present study, we utilized functional magnetic resonance imaging (fMRI) to investigate how brain activity differs between performances that involve ipsilateral and contralateral hand–foot coordinated movements. As mentioned above, brain activities in the SMA, PMA, and cerebellum during ipsilateral hand–foot coordinated movements are higher for the (more difficult) opposite direction movements as compared to those for the (easier) same direction movements (Heuninckx et al., 2005; Van Impe et al., 2009). In addition, ipsilateral hand–foot coordinated movements in the opposite direction are more difficult than those using the contralateral hand and foot. In light of, and to extend these findings, we investigated whether ipsilateral hand–foot coordination would increase activation of the secondary motor areas and cerebellum as compared to that which occurred during contralateral hand–foot coordination for opposite directional movements.

## Materials and Methods

### Subjects

Twenty-one healthy volunteers participated in the experiment (14 males and 7 females; mean age ± standard deviation (SD), 24 ± 2 years). All subjects were right-handed according to the Edinburgh Inventory (Oldfield, 1971), and right-footed according to Chapman’s Footedness Test (Chapman et al., 1987). Before the experiment, written informed consent was obtained from all subjects. The study was approved by the Human Research Ethics Committee of Waseda University.

### Materials

Magnetic resonance images were acquired via a 1.5 T MR scanner (Signa, General Electric, Wisc., USA), using an 8-channel head coil. fMRI data with BOLD contrast were acquired using T2-weighted echo planar imaging free induction decay sequences with the following parameters: TR 3000 ms, TE 50 ms, FOV 22 cm × 22 cm, slice thickness 5 mm and gap 1 mm, flip angle 90°, resulting in a voxel size 4 mm × 4 mm × 5 mm. To pace the frequency of task movement, the subject wore non-magnetic goggles (VisuaStimDigital, Resonance Technology Co., USA) a visual stimulus involving a blinking red-filled circle on a black background which was controlled by a PC and projector system. In addition to the blinking red-filled circle, letters indicating the movement direction the subjects had to perform were presented under the circle during the execution task block. These letters were not displayed during the rest block. The angular displacement of each limb was also measured at 1 kHz using non-magnetic electrical goniometers (S700 MRI Version, MEASURAND). This data was low-pass filtered with a cut-off frequency of 10 Hz. Signals from the displacement of joint angles were converted into digital format with an A/D converter system (Power lab 16/30, ADInstruments, Nagoya, Japan). Signals indicating the joint angle could be checked in real time in a scan room.

### Task

The subjects performed periodic simultaneous movements of the hand (wrist flexion and extension) and foot (dorsiflexion and plantarflexion) in the sagittal plane to a pace set (1.1 Hz) following the visual stimuli described above.

**FIGURE 1:**
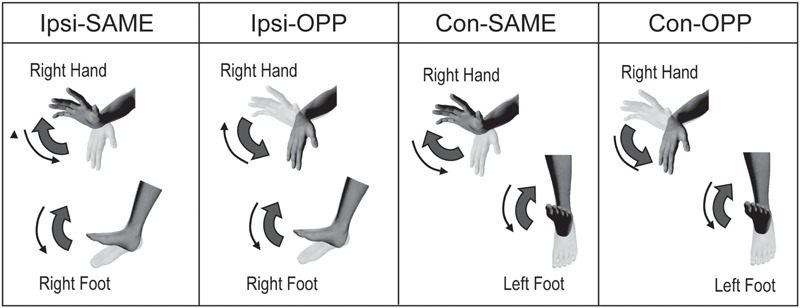
**Experimental task.** Periodic ipsilateral (Ipsi) or contralateral (Con) coordination of the hand and foot during same or opposite (OPP) directional movements.

**FIGURE 2:**
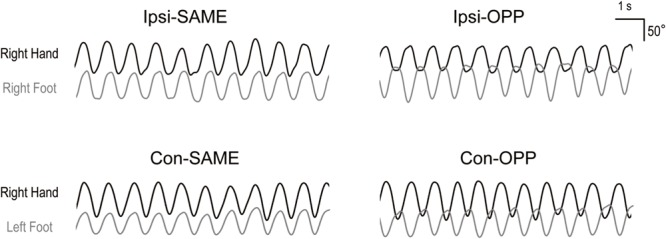
**Representative kinematic traces for the four tasks.** Upper figures indicate ipsilateral hand–foot coordination. Lower figures indicate contralateral hand–foot coordination. Black and gray lines show wrist and ankle angles, respectively.

There were four different tasks. These consisted of two limb combinations (ipsilateral hand–foot: right hand and right foot, contralateral hand–foot: right hand and left foot) and two movement directions (same and opposite direction). These were performed as described in previous studies (Meesen et al., 2006; Fujiyama et al., 2013; Nakagawa et al., 2015). During the tasks, subjects maintained their forearms in a prone orientation. To summarize, there were four tasks: (1) same directional movements of ipsilateral hand and foot (Ipsi-SAME), (2) opposite directional movements of ipsilateral hand and foot (Ipsi-OPP), (3) same directional movements of contralateral hand and foot (Con-SAME), and (4) opposite directional movements of contralateral hand and foot (Con-OPP; **Figures 1** and **2**).

### Procedure

Several days before the fMRI scan, subjects practiced the task. This was necessary in order to avoid phase-transition from the opposite directional movements to the same directional movements. The occurrence of phase-transition could result in different sample numbers between tasks. For the fMRI scans, a 10 min 12 s run was done for each limb combination condition. This involved ten alternate repetitions of the task and included a rest period. Tasks of ipsilateral or contralateral combination were performed in separate scans, and the same and opposite directional movements were alternatively performed in trial blocks. Five trials thus were performed for each task. Half of the subjects began the same directional movements and the other half began with the opposite directional movements. When the red-filled circle with the letters indicating movement direction was presented, the subjects executed the task. When the red-filled circle without any letters was presented, subjects were asked to not move, and relax. The duration of each trial and each rest periods was 30 s. In order to stabilize the magnetic field, the first 12 s of each scan were not used in the analysis. If the waveforms of goniometers indicated that subject’s performance was incorrect during the scan (e.g., a transition from opposite direction to same direction occurred or the movements were not as instructed), this scan session was stopped and restarted from the beginning.

### Analysis

#### Kinematic Analysis

To investigate the performance of two-limb coordination, the relative phase (Φ) between the movements of the hand and foot was calculated for each cycle as Φ*_hf_* = *360°(t_f,i_ – t_h,i_)/(t_f,i+1_ – t_f,i_)*, where *t_h,i_* and *t_f,i_* indicate the time of the *i* th peak extension of the hand and foot, respectively (Carson et al., 1995; Ridderikhoff et al., 2005; Volman et al., 2006; Nakagawa et al., 2013). To evaluate the stability and accuracy of the coordinated movements, standard deviation (SDΦ), and absolute errors (AEΦ) of the relative phases between two limbs were used, respectively. SDΦ was the standard deviation of relative phases in each trial (i.e., 30 s). AEΦ was calculated by averaging the absolute errors in each cycle relative to the target phase (SAME: 0°, OPP: 180°), and we averaged them in each trial (30 s). We also calculated the difference between peak extension/dorsiflexion and flexion/plantarflexion angles for each cycle in order to confirm movement amplitude for the hand and foot, respectively. The mean cycle durations of each task for hand and foot movements were also obtained. For each index, two-way ANOVAs with repeated measures [combination (ipsilateral and contralateral) × direction (same and opposite)] were performed. When an interaction was detected, significant differences between tasks were calculated by the use of paired *t*-tests with a Sidak correction. The statistical threshold for kinematic analysis was set at *p* < 0.05.

#### fMRI Data Processing

The fMRI data were analyzed with a Statistical Parametric Mapping (SPM8, Wellcome Department of Cognitive Neurology, London, UK) program implemented in MATLAB (Mathworks, Sherbon, MA, USA). For each subject, all EPI volumes were realigned, and they were normalized to the standard space of the Montreal Neurological Institute (MNI) brain. The images were smoothed with Gaussian filter of 8 mm full width at half maximum (FWHM). High-pass filters (128 s) were also applied and low frequency noise and global changes in the signals were removed. All statistical analyses were performed in the context of the general linear model on the first analysis. In addition, differences in brain activation between movement direction were investigated by contrasting the two movement direction for each combination [(Ipsi-SAME – Ipsi-OPP), (Ipsi-OPP – Ipsi-SAME), (Con-SAME – Con-OPP), and (Con-OPP – Con-SAME)]. In addition, comparisons between limb combinations were done for the following contrasts: [(Ipsi-OPP – Ipsi-SAME) – (Con-OPP – Con-SAME)], [(Ipsi-SAME – Ipsi-OPP) – (Con-SAME – Con-OPP)], [(Con-OPP – Con-SAME) – (Ipsi-OPP – Ipsi-SAME)], and [(Con-SAME – Con-OPP) – (Ipsi-SAME – Ipsi-OPP)]. To minimize the effects of the head motion artifacts, we included the six head motion parameters as regressors in each run. These motion parameters were estimated in the realignment procedure. Subject-specific contrast images of the estimated parameter were used for a subsequent second-level analysis (random-effect model; Friston et al., 1999). Comparisons between the tasks were evaluated by utilizing a subtraction analysis. The statistical threshold was set at *p* < 0.001 uncorrected. Additionally, we discarded small clusters of less than 10 voxels. If significant activation was found in the white matter, the result was excluded from description in the results section and tables. Anatomical locations were determined utilizing the anatomy tool box (version 1.8) of SPM. Then, in order to evaluate the extent to which the quantitative difference was dependent upon limb combination, we calculated the percentage of BOLD signals of the task based on rest (percent signal change: PSC) for significant clusters that were identified by subtraction analysis for each subject. To evaluate differences in brain activity that were related to the *directional constraint* between ipsilateral and contralateral combinations, we compared the subtraction value for the PSC during the same directional task with that of the opposite directional task for each limb combination (Nakagawa et al., 2015). In addition to the comparison of limb combinations, differences in movement direction were also calculated for each combination. In each case, a paired *t*-test was performed (statistical threshold: *p* < 0.05).

## Results

The kinematic and fMRI data of one subject was removed from the analysis because the subject’s head movement was 12 mm on the *z*-axis (over two times of the size of 1 voxel) during the scan. The remaining 20 subjects were analyzed for both kinematic and fMRI data.

### Kinematic Data (**Table 1**)

#### Relative Phase Indexes

In SDΦ, a two-way ANOVA detected a main effect of direction [*F*(1,19) = 15.51, *p* < 0.001] and an interaction [*F*(1,19) = 6.53, *p* < 0.05]. *Post hoc* tests found a significant difference between Con-OPP and Con-SAME (*p* < 0.05) and between Ipsi-OPP and Ipsi-SAME (*p* < 0.001). In AEΦ, a two-way ANOVA found an interaction [*F*(1,19) = 14.21, *p* < 0.05]. *Post hoc* tests found a significant difference between Ipsi-OPP and Con-OPP (*p* < 0.05).

#### Movement Duration and Amplitude

For cycle duration, a two-way ANOVA detected neither a main effect nor an interaction for each limb. For movement amplitude of the right hand in the four tasks, a two-way ANOVA detected a significant a main effect of direction [*F*(1,19) = 33.83, *p* < 0.001] but no interaction. For the right and left foot, a two-way ANOVA found no main effect nor interaction.

### fMRI Data

#### Whole-Brain Analysis

Regions activated during Ipsi-SAME were located in the left primary motor cortex (M1), primary somatosensory cortex (S1), PMA, SMA, secondary somatosensory area (S2), superior parietal gyrus (SPG), and posterior lobule of cerebellum. In the right hemisphere, the inferior parietal cortex (IPC), S2 and anterior lobule of the cerebellum were activated. In addition, vermis anterior and posterior lobule of cerebellum were activated (**Table 2**).

**Table 1:** Kinematic data.

	Ipsilateral		Contralateral	
	SAME	OPP	SAME	OPP
**Relatetive phase indexs**				
AE ϕ (°)	18.1 ± 7.8	24.7 ± 10.5	19.5 ± 8.1	20.7 ± 9.1
SD ϕ (°)	13.6 ± 4.7	20.0 ± 4.3	15.1 ± 4.2	19.1 ± 4.4
**Amplitude**				
Hand (°)	66.8 ± 31.2	79.4 ± 30.6	63.4 ± 35.5	73.5 ± 35.7
Foot (°)	39.6 ± 12.5	41.5 ± 12.0	37.2 ± 16.7	37.0 ± 14.0
**Cycle duration**				
Hand (ms)	876 ± 12	879 ± 21	871 ± 25	867 ± 25
Foot (ms)	876 ± 12	878 ± 21	870 ± 25	866 ± 25

*AE, absolute error.*

**Table 2:** Brain regions with significant activation in each task.

		Ipsi-SAME				Ipsi-OPP				Con-SAME				Con-OPP			
Region	Side	MNI coordinates			*Z*-score	MNI coordinates			*Z*-score	MNI coordinates			*Z*-score	MNI coordinates			*Z*-score
		*X*	*Y*	*Z*		*X*	*Y*	*Z*		*X*	*Y*	*Z*		*X*	*Y*	*Z*	
**Frontal Lobe**																	
M1	R									12	-28	78	4.95	12	-28	78	4.84
	L	-4	-28	68	6.85	-8	-25	62	7.28	-30	-26	58	5.45				
S1	L	-12	-16	76	5.47	-32	-42	62	4.88	-26	-40	48	3.24	-26	-40	70	5.00
PMA	L	-28	-26	64	6.10	-30	-26	68	4.65	-30	-26	72	5.53	-32	-24	72	4.68
SMA	R									4	-14	72	7.04	2	-22	62	6.26
	L	-4	-18	52	6.10	-4	-14	66	6.69	-6	-8	58	5.64	-6	-8	54	5.52
**Parietal Lobe**																	
S2	R	66	-24	24	3.78	50	-32	18	4.26	42	-26	22	4.27				
	L	-46	-24	18	5.22					-50	-28	20	4.56	-42	-28	18	4.08
SPL	L					-14	-42	68	6.15								
IPC	R	54	-34	28	3.85	58	-34	32	3.62	45	-32	22	4.36	48	-32	22	4.27
	L					-46	-26	18	4.78					-52	-26	18	4.07
STG	L	-54	2	-2	3.52												
**Sub-lobar**																	
Thalamus	R									20	-20	6	3.25				
	L	-20	-20	4	5.35					-18	-20	6	3.35	-14	-18	-2	3.35
Insula	R													36	-26	18	4.33
	L													-46	2	2	3.58
Putaman	R																
	L	-28	0	6	3.57												
**Cerebellum**																	
Anterior lobule	R	10	-52	-18	5.76	10	-52	-14	5.81	12	-50	-20	4.73	20	-46	-26	3.50
	L									-10	-38	-26	4.72	-18	-36	-28	4.32
Posterior lobule	R	-32	-58	-28	3.93	30	-46	-32	3.68	26	-44	-30	3.62	6	-66	-34	3.16
Vermis anterior lobule		4	-60	-12	5.31					-2	-46	-12	4.97	0	-46	-12	4.70
Vermis posterior lobule		4	-68	-42	4.56	6	-68	-38		0	-70	-42	3.89	6	-52	-12	4.67

Brain activities related to Ipsi-OPP were located in the left M1, S1, PMA, SMA, superior parietal lobule (SPL), right S2, anterior and posterior lobule of the cerebellum, bilateral IPC, and vermis posterior lobule (**Table 2**).

Activated areas in the Con-SAME were shown in the left S1, PMA, posterior lobule of cerebellum, bilateral M1, SMA, IPC, S2, thalamus, and vermis anterior and posterior lobule (**Table 2**).

Areas activated by Con-OPP were the left S1, PMA, S2, thalamus, right posterior lobule of the cerebellum, bilateral SMA, IPC, insula, anterior lobule of the cerebellum, and vermis anterior and posterior lobule (**Table 2**).

#### Contrast between Tasks

**FIGURE 3:**
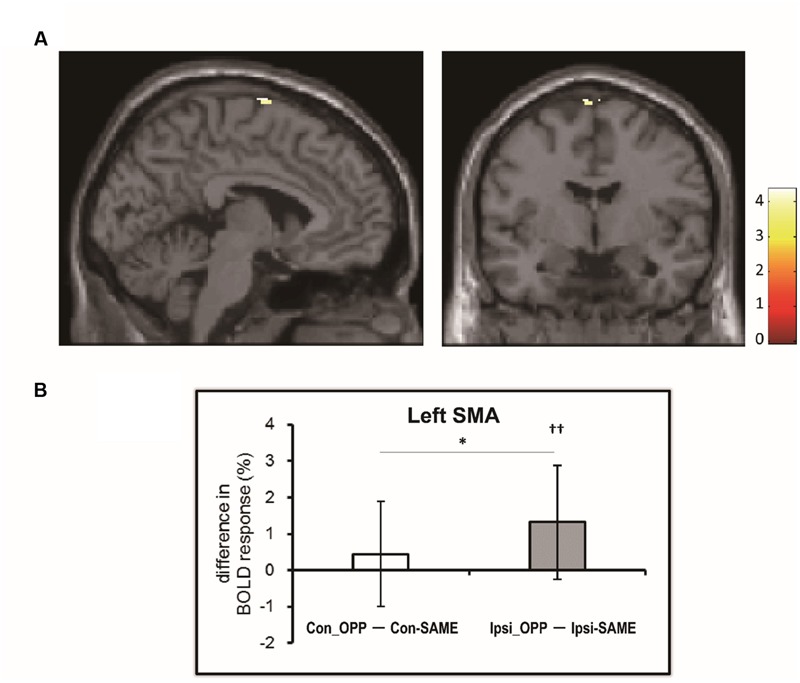
**(A)** The region in which BOLD activity is significantly different between tasks [(Ipsi-OPP) – (Ipsi-SAME)]. **(B)** The difference in percent signal change (PSC) between the opposite and same directional movement (white: contralateral, gray: ipsilateral) in the regions that was observed in the subtraction analysis. Higher numbers indicate stronger activity during the opposite directional movement compared to the same directional movement. Asterisk indicates the significant difference between (Con-OPP – Con-SAME) and (Ipsi-OPP – Ipsi-SAME; ^∗^*p* < 0.05). Daggers indicate that the PSC of (Ipsi-OPP – Ipsi-SAME) was significantly greater than zero (††*p* < 0.01).

We investigated the difference in brain activation between tasks by utilizing a subtraction analysis. No regions showed more activity during Con-OPP vs. Con-SAME while significant additional activation during the Ipsi-OPP compared to the Ipsi-SAME was observed in the left SMA (**Figure 3**; **Table 3**).

**Table 3:** Activated region in direct comparison between tasks.

		Ipsi-OPP – Ipsi-SAME			
Region	Side	MNI coordinates			*Z*-score
		*X*	*Y*	*Z*	
**Frontal Lobe**			
SMA	L	-4	-4	78	3.59

In order to detect the specific regions related to limb combination, we made the following comparison of the subtraction values: [(Ipsi-OPP – Ipsi-SAME) – (Con-OPP – Con-SAME)], [(Ipsi-SAME – Ipsi-OPP) – (Con-SAME – Con-OPP)], [(CON-OPP – Con-SAME) – (Ipsi-OPP – Ipsi-SAME)], and [(CON-SAME – CON-OPP) – (Ipsi-SAME – Ipsi-OPP)]. However, none of the above combination involving the comparison of subtraction values were significant.

#### Region of Interest Analysis (PSC)

We found a significant activation in the left SMA (-4, -4, 78) in the former whole brain subtraction analysis [only in (Ipsi-OPP – Ipsi-SAME)] (**Figure 3A**). In order to quantitatively investigate the BOLD signals in the region, PSCs were compared across tasks. As a result, significant differences in PSC was observed between (Ipsi-OPP – Ipsi-SAME) and (CON-OPP – CON-SAME*p* < 0.05) and between Ipsi-OPP and Ipsi-SAME (*p* < 0.001; **Figure 3B**).

## Discussion

In the present study, we investigated how brain activity differed in relation to differences in performance of ipsilateral and contralateral hand–foot combinations. We paid special attention to the “directional constraint.” This concept highlights the observation that there is a tendency for simultaneous coordinated movements to be more unstable and inaccurate when performed in the opposite direction as compared to performing in the same direction. Kinematic data suggest that (1) both ipsilateral and contralateral hand–foot coordination show a higher variability in opposite directional movements as compared to same directional movements and (2) opposite directional movements of the ipsilateral limbs are less accurate than those of the contralateral limbs (**Table 1**). Thus, under the present experimental conditions the magnitude of the directional constraint was larger for the ipsilateral combination than for the contralateral combination. These results are consistent with previous studies (Kelso and Jeka, 1992; Swinnen et al., 1995; Hiraga et al., 2004; Nakagawa et al., 2015).

An fMRI whole-brain analysis demonstrated activity in the left M1, left S1, left SMA, left PMA, right and left IPC, right and left S2, and right and left cerebellum for the ipsilateral hand–foot combination task (**Table 2**). Activity in these regions has been seen consistently in a number of studies that utilized a model involving ipsilateral hand–foot coordinated movements (Ehrsson et al., 2000; Debaere et al., 2001; Heuninckx et al., 2005, 2008; Rocca et al., 2007). The M1 and SMA were activated in both hemispheres in the contralateral task. This was likely due to the bilateral use of the limbs in the contralateral task.

Although the cerebellum has been regarded as an important region in interlimb coordination, especially when executing the opposite directional movement (Debaere et al., 2001; Rocca et al., 2007), there was no difference in activity in the cerebellum between ipsilateral and contralateral hand–foot coordination. This indicates that activity in the cerebellum during hand–foot coordination was similar among the various conditions. In addition, in the present study, there was no difference in cerebellum activity between the same and opposite directional movement for both ipsilateral and contralateral combinations. This finding is in conflict with that of previous studies (Debaere et al., 2001; Rocca et al., 2007). It is generally accepted that the cerebellum plays a role in correcting movement error (Ramnani, 2006). The lack of a difference in cerebellar activity seen in our study might be due to the fact that there were less errors during the tasks with a lower frequency (1.1 Hz) as compared to those in a previous study (2.0 Hz: Nakagawa et al., 2015). In any case, function of the cerebellum in interlimb coordination would differ from that of the SMA.

### Difference in Activity in the SMA Depending on Limb Combination

Activation of the SMA was observed in all tasks (**Table 2**). It has been suggested that there is an extra activation of the SMA during any interlimb coordination task as compared to a task involves only a single limb movement (Debaere et al., 2001). This finding has led to the conclusion that the SMA has a role in the coordination of multi limb movements. On the other hand, subtraction analysis indicated that there was greater activity in the SMA during the Ipsi-OPP condition as compared to the Ipsi-SAME condition (**Table 3**; **Figure 3A**). This is consistent with an early MRI study in which brain activity associated with the ipsilateral hand–foot coordination was investigated (Debaere et al., 2001). This result implies that activity in the SMA is greater during opposite directional movements than in same directional movements for the ipsilateral combination. In addition, this effect would be greater in the ipsilateral combination than that in the contralateral combination. In order to quantify the amount of difference in activity in the SMA, we also calculated the PSC. The results showed that (1) a difference in SMA activity that was dependent upon movement direction was only confirmed in the ipsilateral limb combination, but not in the contralateral one (**Figure 3B**). (2) The PSC of the SMA during opposite directional movements as compared to same directional movements was higher in the ipsilateral limb combination than in the contralateral one (**Figure 3B**). Thus there was a greater BOLD response in the SMA during ipsilateral hand–foot coordinated movements in an opposite direction. Thus these differences in SMA activity between ipsilateral and contralateral combination suggested that SMA is involved in difference in behavioral performance depending on limb combination.

### No Difference in Activity in the PMA Depending on Movement Direction

Previous studies have demonstrated that corticospinal excitability of the resting upper limb muscles is modulated depending on the cyclic movement phase of the ipsilateral lower limb in a way that facilitates same directional movements of the ipsilateral hand and foot (Baldissera et al., 2002; Borroni et al., 2004). In addition, Byblow et al. (2007) proposed that the PMA is essential in generating the corticospinal modulation which facilitates same directional movements for ipsilateral hand–foot coordination. However, in the present study, activity in the PMA was not different between the same and opposite directional movements in either the ipsilateral or contralateral combinations. This finding suggests that activation of the PMA facilitates same directional movements even in the ipsilateral hand–foot coordination. This would be expected to occur not only for movements in the same direction but also for those in the opposite direction. Indeed, the opposite directional movements strongly tend to be entrained to the same directional movements (Baldissera et al., 1982).

On the other hand, for the contralateral hand–foot coordination, neural modulation that would facilitate same directional movements may not exist or be very weak, because corticospinal modulation of the relaxing forearm muscles which was dependent upon movement phase of the contralateral foot was not observed during movement (Van Den Berg et al., 2011). Therefore, the PMA might not only facilitate the same directional movements during the same directional movement condition but also facilitate opposite directional movements condition during the contralateral combination. Thus, it might be expected that no difference in the subtraction analysis involving (Con-OPP – Con-SAME) would occur. This could be why the PMA was not associated with a difference in the directional constraint in the two combinations. Additionally, it has been considered that the left PMA is critically involved in the temporal processing of cyclic motor tasks (Pollok et al., 2008; Bijsterbosch et al., 2011). This may be the reason the left PMA is commonly activated for all four of the tasks (**Table 2**).

### Possible Role of the SMA in the Ipsilateral Combination

What, then, does the greater activation of the SMA reflect? The SMA has a role of inhibiting unintentional movements during voluntary motor control (Sumner et al., 2007; Wardak, 2011) that extends beyond that of simply coordinating multi limbs (Debaere et al., 2001). One possibility for explaining the higher activity seen for the opposite direction of ipsilateral hand–foot coordination was that the SMA prevented phase-transitions for the same directional movements that were likely induced by signals from the PMA. Previous studies indicate that there is the causal relationship of the SMA function and performance of bimanual coordination in less stable anti-phase (the pattern in which homologous muscles are alternatively activated) movement. For example, functional disturbance of the SMA by double pulse or repetitive transcranial magnetic stimulation induced behavioral disruption or transition from the anti-phase movement to the stable in-phase movement (the pattern in which homologous muscles are simultaneously activated) (Meyer-Lindenberg et al., 2002; Steyvers et al., 2003). In addition, an increase of SMA excitability induced by anodal transcranial direct current stimulation improves the bimanual anti-phase movement (Carter et al., 2015). Thus, it would be likely that the SMA inhibits neural circuits that induce in-phase movement. As mentioned above, while neural modulation that would facilitate same directional movements would work strongly for ipsilateral combinations (Baldissera et al., 2002; Borroni et al., 2004; Byblow et al., 2007), it would not work (or work only weakly) for contralateral combinations. Indeed, corticospinal modulation of relaxing forearm muscles was not observed during contralateral foot movements (Van Den Berg et al., 2011). Thus, the neural circuits involved with enhancing movements of the hand and foot in the same direction would be stronger for the ipsilateral combination than for the contralateral one. Then additional activity in the SMA would be needed for opposite directional movement in the ipsilateral hand–foot combination in order to prevent a phase-transition.

If inhibition of phase-transition for the ipsilateral hand–foot coordination is necessary on a constant basis, then paying attention to kinesthetic afferent information would be necessary to detect a coming of transition. This would correspond to the notion that the execution of opposite directional ipsilateral hand–foot coordination requires more attention than that of contralateral hand–foot coordination (Hiraga et al., 2004). This is supported by the observation that brain regions involved in attention to sensory signals such as S2 and IPC (Chen et al., 2010; Lee et al., 2013) were activated more in ipsilateral hand–foot coordination in the present study, though the statistical difference did not reach a significant level.

### Limitation of the Present Study

The present study has two major limitations. First, we did not investigate all limb combination for the four limbs. Subjects only performed the limb combination of “right hand and right foot” and “right hand and left foot.” Thus it remains unclear whether laterality (right hand or left hand) influenced brain activities that were dependent on limb combination. Therefore, in the future, it will be important to clarify the effect of laterality by utilizing a task including left hand in order to generalize our findings. In relation to this, the present study demonstrated a difference in “left” SMA activity between the ipsilateral and contralateral combination. This might be due to the fact that we compared brain activities of the ipsilateral and contralateral combinations only with the “right” hand as a common body part. Therefore, further investigation utilizing the “left” hand as a common body part will be required in order to examine whether activity in the left SMA reflects a dominance of the left side or is simply due to movements of the right hand.

Second, we set a lower movement frequency in this study (1.1 Hz) than in our previous study (2.0 Hz; Nakagawa et al., 2015) in order to prevent phase-transition. This might explain why we observed a smaller effect of limb combination in the present study. In a future study, it would also be instructive to examine the effect of a wide range of movement frequencies as well as the effect of laterality in order to determine the generality of the present findings.

## Conclusion

The objective of the present study was to investigate how brain activity is related to the performance of periodic hand–foot coordination under different limb combinations. The results suggested that there is a greater activation of the SMA in the ipsilateral combination as compared with the contralateral combination.

## Author Contributions

KN, SK, NM, and KK designed research. KN, SK, and NM conducted the experiments. KN, SK, and NM analyzed data. KN, NM, and KK wrote the manuscript.

## Conflict of Interest Statement

The authors declare that the research was conducted in the absence of any commercial or financial relationships that could be construed as a potential conflict of interest.
